# A plasma proteomics method reveals links between ischemic stroke and MTHFR C677T genotype

**DOI:** 10.1038/s41598-017-13542-6

**Published:** 2017-10-17

**Authors:** Zhenchang Zhang, Qi Yan, Jia Guo, Xueping Wang, Wei Yuan, Lei Wang, Lixia Chen, Gang Su, Manxia Wang

**Affiliations:** 1 Department of Neurology, the Second Hospital of Lanzhou University, Lanzhou, 730030 China; 2 School of Basic Medical Sciences, Lanzhou University, Lanzhou, China

## Abstract

Methylene Tetrahydrofolate Reductase (MTHFR) catalyzes the conversion of methylene tetrahydrofolate to methylte trahydrofolate. The 677th nucleotide of the MTHFR gene is often regarded as a risk factor of cardiovascular disease. Previous studies demonstrated an elevated risk of ischemic stroke with the MTHFR677TT genotype. In this study, we employed a plasma proteomics method to investigate the connection between the polymorphism of the target nucleotide and stroke. In total, 28 protein spots were differentially expressed between the two groups, and of which, 25 protein spots were up-regulated and 3 were down-regulated. Five randomly selected spots were successfully identified as Haptoglobin (HPT) and Transferrin (TRFE). A functional analysis indicated that most of the differential expressed proteins (DEPs) were related to the inflammatory immune response. A Kyoto Encyclopedia of Genes and Genomes (KEGG) pathway analysis showed that these DEPs were involved in the complement cascade reaction. Meanwhile, protein-protein interactions (PPIs) analysis highlighted the novel association between the C677T MTHFR genotype and Vitamin D binding protein (DBP), which was confirmed by a molecular genetic analysis. The results suggested that the phenotype of the MTHFR might be associated with multiple proteins that have a synergistic effect, which might be related to the mechanism of ischemic stroke.

## Introduction

Numerous polymorphisms have been identified after the completion of the sequencing of the human genome^1^. However, among these polymorphisms, the phenotypic effects in cells are largely unknown, including how the polymorphisms impact physiological function, whether they are associated with diseases and so on. There is a common polymorphism in the gene encoding the catalytic domain of methylene tetrahydrofolate reductase (MTHFR), namely there is a C-T substitution at position 677 of the gene sequence, which leads to an alanine to valine switch^2–4^. The polymorphism of the 667th nucleotide in MTHFR geneis regarded as a risk factor for cardiovascular disease (CVD)^5,6^. This polymorphism leads to splice site changes, decreased mRNA stability and changes of the enzymatic protein conformation, which leads to a reduction or loss of MTHFR activity, thereby increasing the level of homocysterine (HCY) in the plasma^7^. While the latter is an independent risk factor for obstructive peripheral arterial diseases, such as cardiovascular, cerebrovascular and peripheral diseases and it is also an independent risk factor for venous thrombosis^8^. Decreased enzyme activity is caused by the MTHFR genetic polymorphism, and this is a potential risk factor for the progression of hyperhomocysteinemia, but the relationships are still controversial^7–9^.

A proteomic analysis of plasma is a relatively practical snapshot tool to compare and analyze the expression atlas under different physiological and pathological genetic statuses^10^. This technology provides an opportunity to further understand disease pathogenesis, screen for biomarkers and early diagnosis and treatment^11^, especially, when we focus on the study of the overall proteins’ expression and the relationship between the genotype and phenotype^12^.

Epidemiologic studies have revealed that a high risk of venous thrombosis is associated with the MTHFR 677TT genotype and arterial stroke^13^. However, whether this polymorphism directly participates in the progression of CVD remains unclear. In this study, we used plasma proteomics to examine whether stroke patients with the C677T polymorphisms show obviously changes in protein expression. This analysis can gives some hints to explain the occurrence of stroke.

## Results

### Establishment of the differential protein expression spectrum

**Figure 1:**
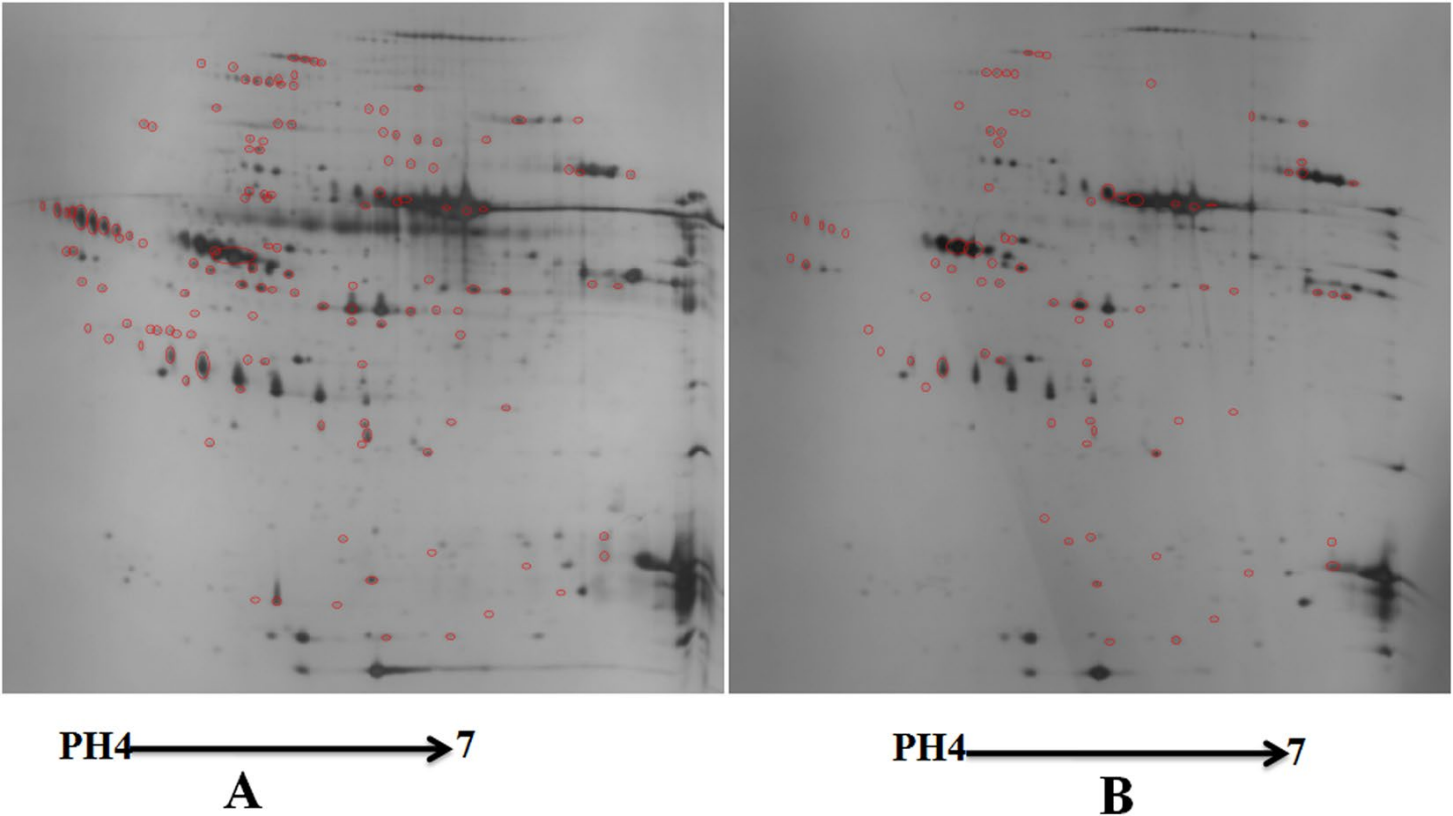
Results of the 2D-PAGE reveal the profiles of differential expression proteins (DEP) between the C/C genotypes and T/T genotypes. The red circles in the figure are the differential protein spots. A means the T/T genotypes and B means the C/C genotypes.

This study used 2D-gel electrophoresis to analyze plasma proteins in ischemic stroke patients with the MTHFR genotypes C/C and T/T. The results of the counts between the groups were 259 ± 12 and 195 ± 17, respectively. The mean match ratio was 84.75%, and the correlation coefficient was 77.9% (>70%). A total of 137 different spots were found after the comprehensive comparison (Fig. 1). The correlation coefficient of the scatter diagram was 0.78 (>0.4), and the reproducibility was consistent among the groups (Supplementary Figure S1). Twenty-eight proteins were selected that had significant differences that were greater than double. Moreover, the differential expression proteins (DEPs) were the same between each of the repeats. (Supplementary Figure S2). The expression of 25 protein spots was up-regulated, while 3 protein spots were down-regulated in the mutant (TT) individuals (Table 1).

**Table 1:** Differential expression proteins (DEP) between the C/C genotypes and the T/T genotype.

PDQuest No. (SSP)	Accession No. SWISS-PROT	Protein name	Gene name	pI	Mw (KD)	Fold change*
0515	P01011	AACT	SERPINA3	4.73	61.591	>10
0609	P01011	AACT	SERPINA3	4.70	60.696	>10
0605	P01011	AACT	SERPINA3	4.70	62.603	>10
1401	P02678	FIBG	—	5.07	51.347	8.84
1402	P02678	FIBG	—	5.13	51.207	4.31
2303	P02678	FIBG	—	5.34	49.566	2.23
4302	P02678	FIBG	—	5.56	48.107	2.01
3304	P02678	FIBG	—	5.44	48.107	3.47
0513	P02765	FETUA	AHSG	4.61	56.313	>10
3208	P02766	TTHY	TTR	5.52	35.391	3.72
0507	P01009	A1AT	SERPINA1	4.99	53.626	3.42
0505	P01009	A1AT	SERPINA1	4.95	54.065	2.36
1809	P00450	CERU	CP	5.20	123.705	4.33
2810	P99003	IGSA	—	5.23	93.722	4.13
0203	P25311	ZA2G	AZGP1	4.91	40.977	3.31
0301	P00738	HPT	HP	4.88	44.344	2.68
2205	P00738	HPT	HP	5.38	38.749	2.46
1803	P00450	CERU	CP	5.09	126.365	2.18
2705	P08697	A2AP	SERPINF2	5.17	66.097	>10
0802	P09871	C1S	C1S	4.81	88.849	>10
0803	P09871	C1S	C1S	4.83	88.173	>10
5202	P99004	NA3	—	5.77	37.824	2.68
1806	P00734	THRB	F2	5.09	80.154	2.65
0304	P27169	PON1	PON1	4.84	45.937	2.56
4101	P02743	SAMP	APCS	5.55	26.280	2.32
2501	P02774	VTDB	GC	5.24	53.918	0.43
8703	P02787	TRFE	TF	6.50	79.545	0.35
1302	P06727	APOA4	APOA4	5.16	43.627	0.20

Note: ^*^Indicates the ratio of the protein expression level of the C/C genotypes/T/T genotypes.

### Mass spectrum identification results of the differential protein spots

**Figure 2:**
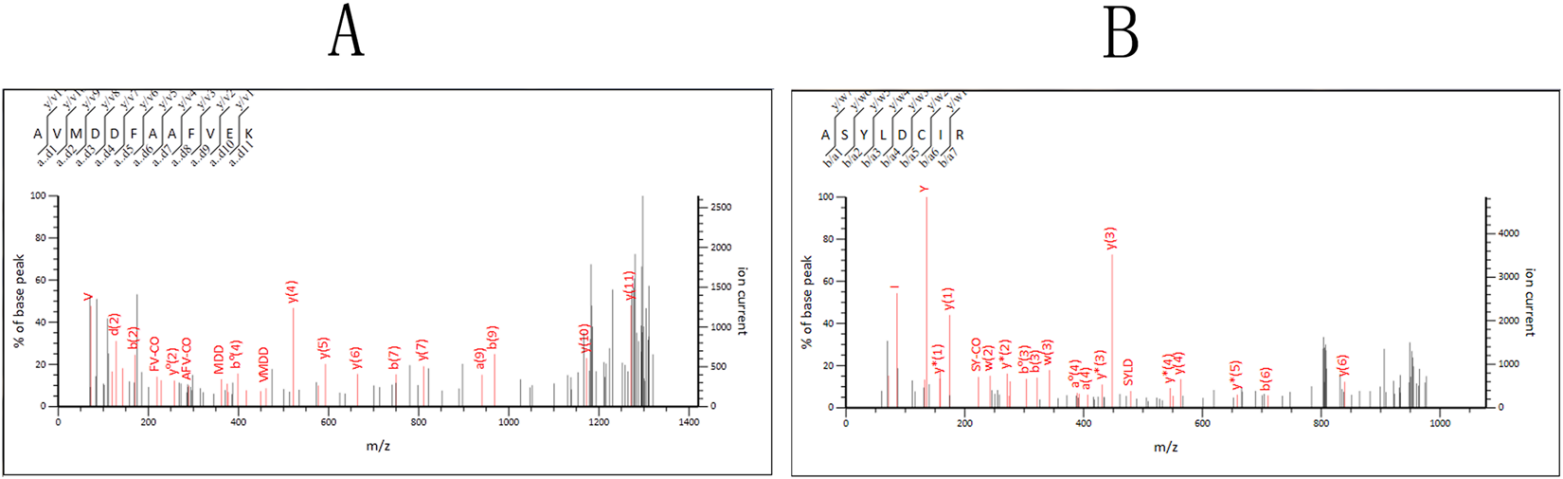
Identification of two randomly selected differential expression proteins (DEP) by using MALDI-TOF-MS (MS/MS) spectrum for parts of the peptides of these proteins.

Four protein spots and 3 serum albumin spots (as controls) were selected from the 28 protein spots for analysis using the SWISS-2DPAGE database to identify the spectrum using matrix assisted laser desorption/ionisation-time of flight mass spectrometry (MALDI-TOF MS) after extracting, digesting and performing enzymolysis on the spots. In total, 5 spots were successfully identified as two types of proteins via the Mascot Wizard software (Table 2). Using the PDquest software, the peptides of protein spot # SSP0301 were analyzed by MALDI-TOF-MS (Fig. 2A). Peptide 6 matched with the protein haptoglobin (HPT). The coverage rate of the amino acids was 14%. Moreover, the experimental relative molecular mass of peptide 6 and its theoretical relative molecular mass were exactly matched. The Mascot score was 212 (Mascot value over 67 means p < 0.05), which revealed that the MS identification results were significant and reliable (P < 0.05). Additionally, using the PDquest software, the peptides of protein spot SSP8703 were analyzed by MALDI-TOF-MS (Fig. 2B). Peptide 8 matched with the protein transferrin (TRFE). The coverage rate of the amino acids was 16%. Moreover, the experimental relative molecular mass of peptide 8 and its theoretical relative molecular mass were exactly matched. The Mascot score was 632, which revealed that the MS identification results were significant and reliable (P<0.05).

**Table 2:** Identify the differential expression proteins (DEPs) by MALDI-TOF-MS.

PDQuest No. (SSP)	Accession No.	Protein name	pI	Mw(KD)	Score	Matches
0301	P00738	HPT	6.13	45.861	212	6(6)
8703	P02787	TRFE	6.81	79.294	632	8(8)

### Bioinformatics analysis of the differentially expressed proteins (DEPs)

**Figure 3:**
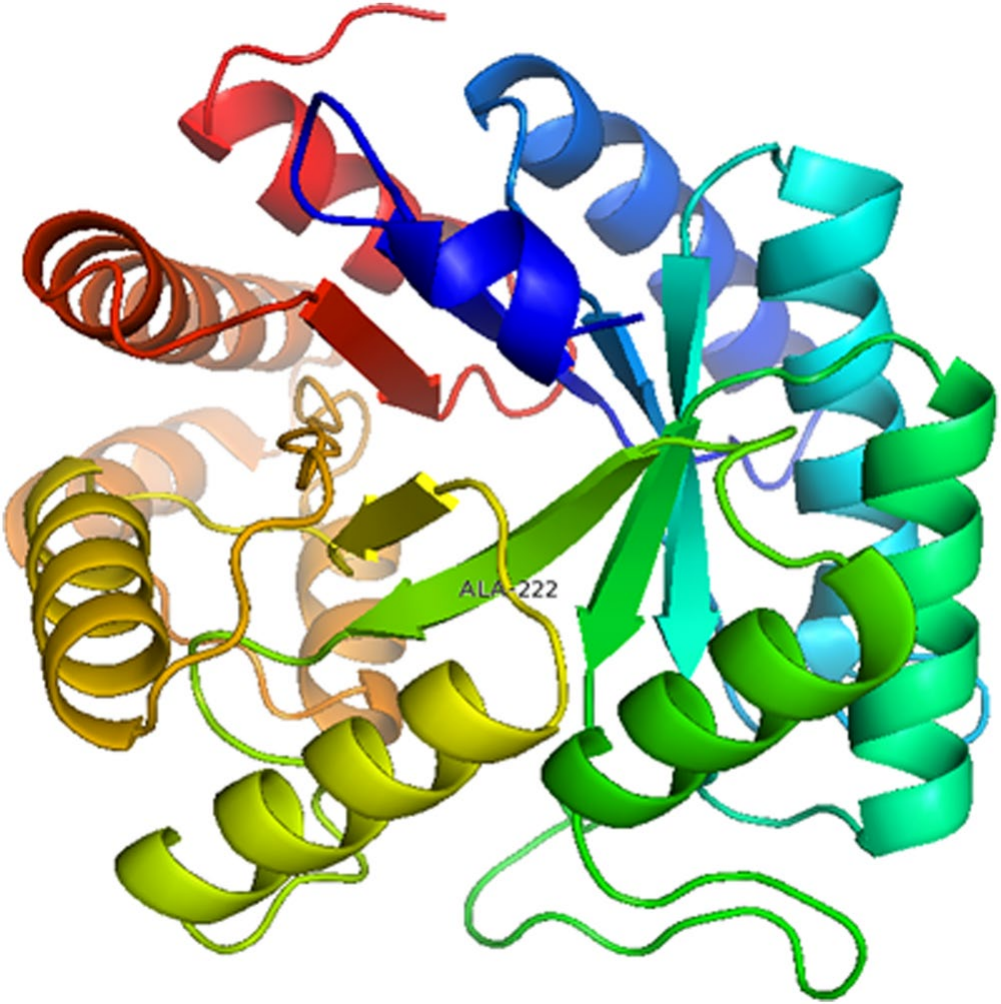
The ensemble of the selected structures is in stereo view. The figure was produced using the Swiss-model and pyMOL programs.

Figure 3 shows the 3D structure of the human MTHFR protein based on similarity to the 3apy.1.A domain (Protein Data Bank (PDB) code: 3apy.1.a), and the target amino acid is labeled and the region presented is Ays48–Thr340.

**Figure 4:**
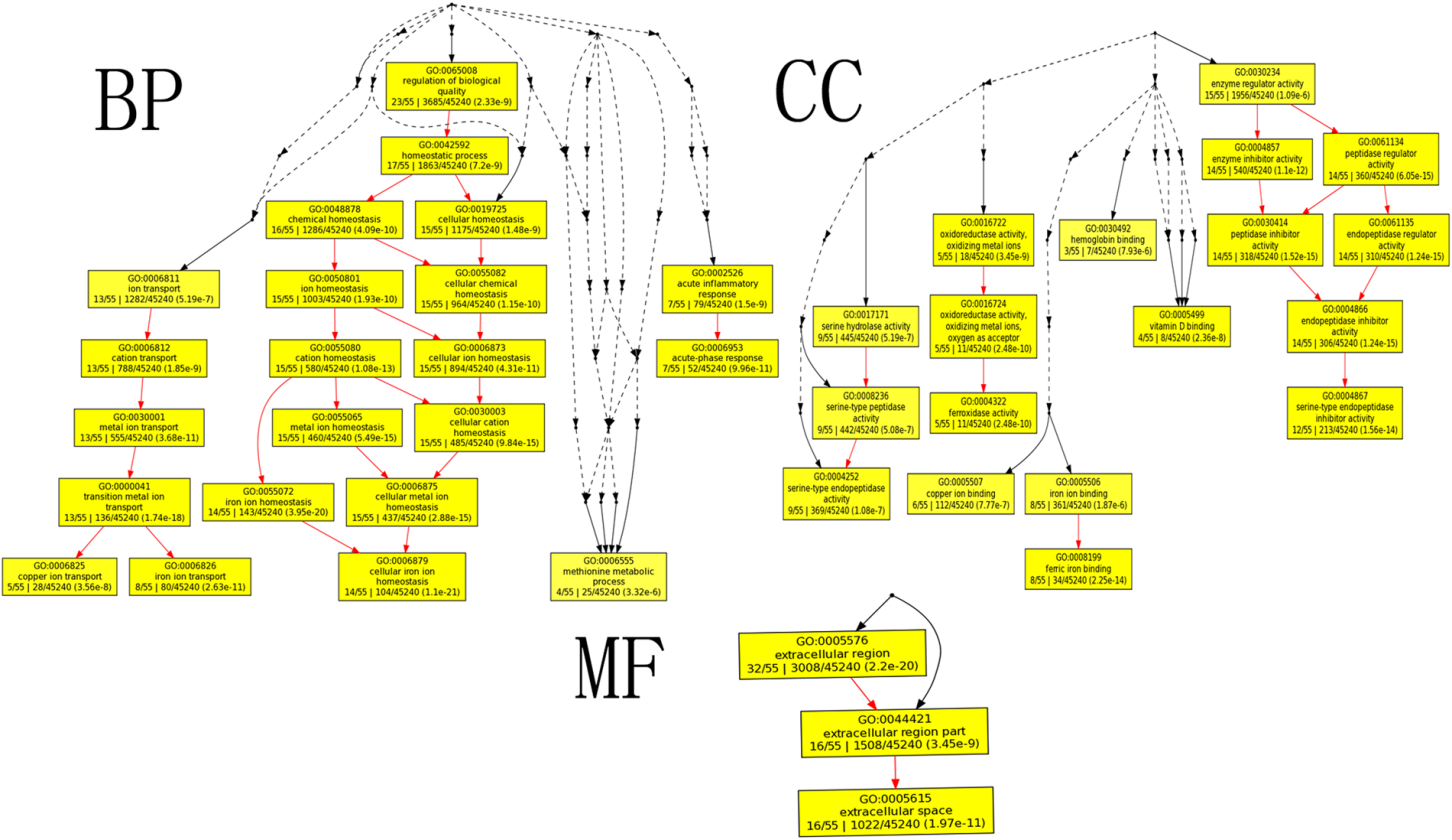
The graph displays enriched GOIDs of 16 seed-specific genes and their hierarchical relationships in “biological process (BP)”, “cellular component (CC)” or “molecular function (MF)” GO categories. Boxes represent GO terms, term definition, p-value and detail information. Significantly enriched GO terms are marked yellow. The degree of color saturation of each node is positively correlated with the significance of enrichment of the corresponding GO term. Non-significant GO terms within the hierarcical tree are shown as points. Branches of the GO hierarchical tree without significant enriched GO terms are not shown. Edges stand for connections between different GO terms. Red edges stand for relationship between two enriched GO terms, black solid edges stand for relationship between enriched and unenriched terms, black dashed edges stand for relationship between two unenriched GO terms.

A GO (gene ontology) enrichment analysis was performed using GOEAST (http://omicslab.genetics.ac.cn/GOEAST/index.php). For an in-depth study, this website is convenient to use, and the data can be displayed as graphical result charts, which provide intuitive data representation. To obtain an overview of the differential expression proteins (DEPs) involved in this study, all 16 identified DEPs were used to perform a GO annotation and an enrichment analysis; the GO annotation results are listed in Supplementary Table S1. The biological processes, cellular components, and molecular functions are shown in Fig. 4. From the perspective of biological processes, homeostasis (GO: 0006879, FDR: 1.1e-21) and transition metal ion transport (GO: 0000041, FDR: 1.74e-18) were the top two significantly enriched terms. From a cellular component perspective, endopeptidase inhibitor activity (GO: 0004866, FDR: 1.24e-15) was the top significantly enriched term. Ferric iron binding (GO: 0008199, FDR: 2.25e-14) was also significantly enriched. From the molecular function perspective, the extracellular region (GO: 0005576, FDR: 2.2e-20) was the top significantly over-represented term.

**Figure 5:**
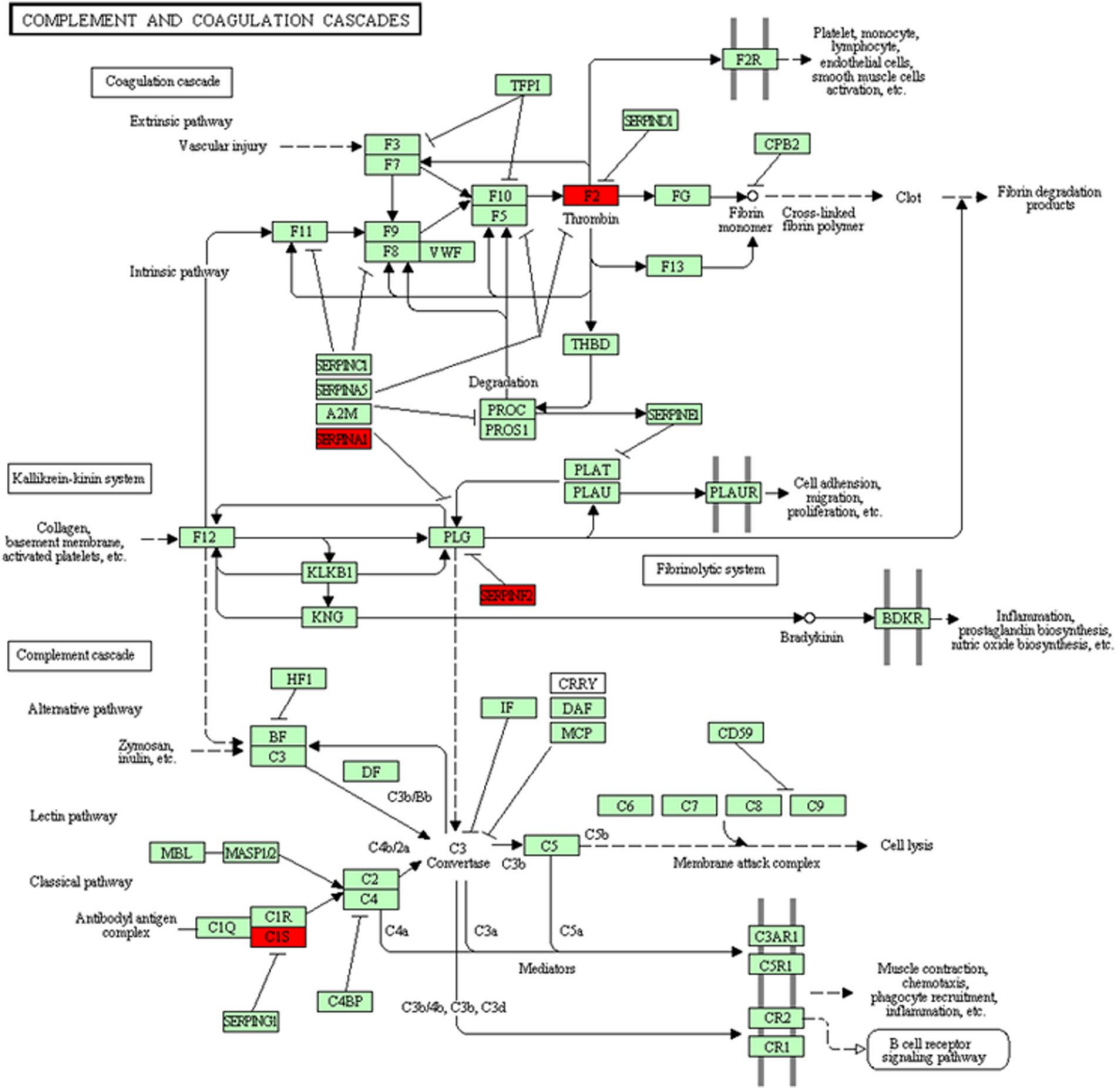
Pathway mapping of the identified DEPs in the KEGG Homo sapiens pathway database. These DEPs were used to search the KEGG Homo sapiens pathway database and were mapped to the Homo sapiens specific pathways by using KEGG Mapper^15–17,38^. In this map, the objects with the red foreground color represent the DEPs, the objects with the green background represent the total proteins in the Homo sapiens KEGG database.

**Figure 6:**
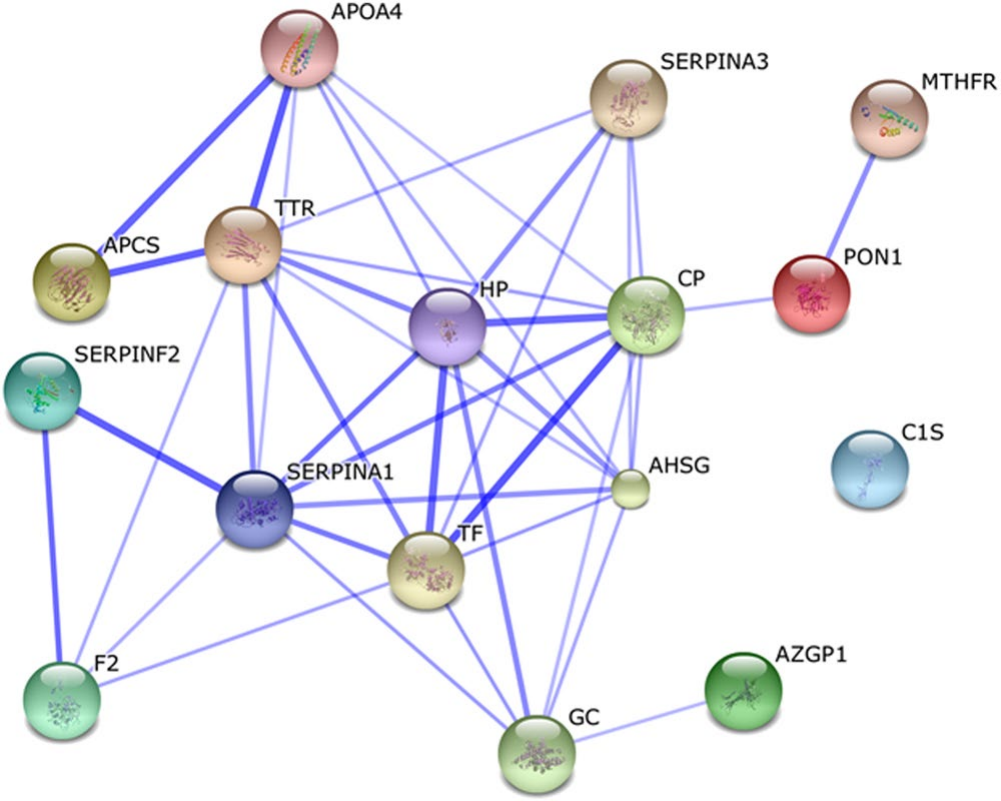
Protein–protein interaction network of MTHFR protein and the identified differential expression proteins (DEPs). The stronger associations are represented by the thicker lines.

Upon further molecular function analysis, sixteen proteins were classified in 10 categories (DAVID, https://david.ncifcrf.gov/home.jsp)^14^. The enrichment score was more than 6.02 (>1.3). The results showed that the biological processes were mainly involved in the acute-phase response and the inflammatory response (Supplementary Table S2). Moreover, four differential expression proteins (DEPs), including F2, SERPAN1, SERPANF2, andC1S, were found to be involved in the cascade reaction of the complement system via the KEGG analysis (Fig. 5)^15–17^. In addition, to identify the interactions between these differential expression proteins, a protein-protein interactions (PPIs) analysis was conducted by using STRING (http://string-db.org/). All of the 16 differential expression proteins were used to construct the interaction network (Fig. 6). To improve the coverage of the PPIs analysis, the confidence level (score) was set without a threshold. SERPINA1, TTR, and HP resided in the central part of the network and were both connected to each other, suggesting the crucial roles of these differential expression proteins (DEPs) in the whole network. Additionally, the MTHFR protein can build the connection to this network through the PON1 protein, which indicates that the MTHFR protein may be a kind of inducing factor that affects the entire cascade network. Furthermore, the C1S protein had no relationship with the other differential expression proteins (DEPs), which suggested that this protein works independently and/or with other connections with this network, which have not been deciphered.

## Discussion

The C677T polymorphism in the gene MTHFR is often associated with cardiovascular diseases (CVD) because it can directly affect folic acid metabolism and also enhance the level of plasma homocysteine, which are considered independent risk factors for ischemic stroke, thrombosis, CVD, and venous thrombosis^7^. In this study, we identified different types of proteins that are expressed in the plasma of stroke patients with the C677T polymorphism.

The differentially expressed proteins mentioned above were mainly involved in the immuno-inflammatory response and the complement cascade. This finding is consistent with previous research studies^18^. For example, NF-nB activation experiments in cultured cells were associated with increased MTHFR mRNA, and the co-transfection of NF-nB and the promoter constructs demonstrated an MTHFR up-regulation by at least 2-fold through its downstream promoter in Neuro-2a cells^18^. Inflammation plays an important role in the formation of atherosclerosis, and numerous evidences revealed that the occurrence and development of atherosclerosis are associated with an inflammatory reaction mechanism^19,20^. Moreover, long-term mild inflammation is also a vital risk factor for ischemic stroke and transient ischemic attack^21^. The current study provides evidence that proteins that participate in the inflammatory reaction are related to stroke and are linked to the MTHFR polymorphism. For example, we discovered that the HPT protein played an important role in the interaction with other differentially expressed proteins. Previous studies indicate that HPT is closely associated with cardiovascular and cerebrovascular diseases. For example, Kiga C., *et al*. found that HPT was a candidate marker for stroke in spontaneously hypertensive rat models^22^. Holme I., *et al*. examined 342,125 patients that died in Swedish hospitals over the course of 11 years and found that high levels of HPT were a significant predictor of ischemic stroke^23^. Additionally, Brea D., *et al*. found that the levels of HPT and amyloid plaque were higher in patients with atherosclerotic stroke than cardiogenic stroke. The evidence previously presented indicates that HPT is an important biomarker in the diagnosis of atherosclerotic stroke^24^.

Moreover, Chen R., *et al*. found that HPT, thyroxine carrier protein, and complement C3 significantly changed after partial cerebral ischemia/reperfusion via studying plasma proteomics in ischemic stroke models^25^. In the protein-protein interaction analysis, HPT and thyroxine carrier protein were identified as predicted functional partners with a score of 0.751. Similarly, in the interactive network, both of these proteins were predicted to provide a basis for participating in the inflammatory and immunologic process in the occurrence of stroke.

As a vital inflammatory mediator, the complement system plays an important role in the occurrence and development of atherosclerosis and ischemic stroke^26^. Many studies reveal that almost all of the complement inherent ingredients and the mRNA expression of various complement regulatory proteins are found in atherosclerosis lesions^27^. Fibrinogen (Fg) plays an important role in the complement system. For example, the generation of thrombin is regarded as a critical step in the coagulation cascade, since thrombin mediates the functions that lead to the formation of blood clots by the cleavage of fibrinogen and factor XIII and the activation of platelets. Previous studies revealed that Fg is an important risk factor for coronary heart disease (CHD), stroke and peripheral angiopathy^28^. For patients with atherosclerosis, the expression of Fg protein was significantly higher than in patients without atherosclerosis in ischemic cerebrovascular disease. In addition, the expression of Fg protein is related to the severity of the disease^29,30^. This study revealed that Fg protein is differentially expressed in the plasma of the patients with the different genotypes.

In the KEGG analysis, four differentially expressed proteins, includingSERPINF2, SERPINA1, F2, and C1S, participated in the complement response process. Individual SERPINF2 and SERPINA1 protein expression may increase with the MTHFR polymorphism, which may suppress plasminogen via the coagulation system, promoting endothelial cells to bind Fg and accelerate platelet aggregation, resulting in thrombosis. Hamzi K., *et al*. performed a meta-analysis, which revealed that prothrombin and MTHFR were risk factors in Indian patients with ischemic stroke^31^. CIS protein is important for the complement cascade and plays a role in the regulation of immune adhesion^32^.

Furthermore, we identified Vitamin D binding protein (DBP) in the PPIs analysis, which is a serum-rich protein that releases actin into the peripheral circulation after cellular damage. In individuals with the MTHFR polymorphism, decreased DBP lowers the affinity of vitamin D, resulting in weakening of the vascular smooth muscles. DBP alterations in MTHFR polymorphisms may have an impact on other functions of DBP^33^. Plasma lipid metabolism disorder is usually associated with cerebrovasular disease. In this study, we found a quantitative reduction of Apolipoprotein A-4 in the mutant individuals. Apolipoprotien is a lipid binding protein that is involved in lipid transport that can either activate or suppress enzymes in the lipoprotein metabolic process or act as a receptor signal in liver cells^34^. We speculate that the individuals with MTHFR polymorphisms may be linked with stroke through Apolipoprotein A-4.

In conclusion, these results revealed that the phenotype of the MTHFR genotype might be associated with multiple proteins, which may have synergistic effects. Moreover, this study also provides the significant differentially expressed proteins between the two groups, which will contribute to understanding the mechanism of ischemic stroke in patients with the MTHFR polymorphism.

## Materials and Methods

### Sample collection and grouping

Plasma from six groups of ischemic stroke inpatients was collected at the Department of Neurology of The Second Hospital of Lanzhou University between September 2010 and May 2012. All of the patients were unrelated Han Chinese male patients between the ages of 60–70 years old. Stroke was confirmed based on the results of a strict neurological examination, CT, or MRI according to thesuggestions of the International Classificationof Diseases (9th Revision, codes 430 to 438). Meanwhile, stroke was defined as a sudden onset of anonconvulsive and focal neurological deficit persisting for >24 hours. We used a GoldMag-Mini Whole Blood Genomic DNA Purification Kit (GoldMag Co. Ltd. Xi’an City, China) to extract DNA from whole blood. Extracted DNA was quantified using NanoDrop2000. Genotyping was done with the Sequenom MassARRAY RS1000 system using the standard protocol recommended by the manufacturer. Data were managed and analyzed using Sequenom Typer 4.0 Software. Genotype was analyzed using a Sequenom MassARRAY RS1000 system and the standard protocol recommended by the manufacturer. In total, 20 patients with the TT geneotype in the C667T sites and 20 patients with the CC genotype in the C667T sites were used as experimental group and control group in this study (Table 3). The study was approved by the Ethics Committee of the Second Hospital of Lanzhou University. The Ethics Committee also approved the related screening, treatment, and data collection from these patients based on the experimental design and analysis of clinical outcome. The methods were carried out in accordance with the approved guidelines and were performed according to the Declaration of Helsinki. All of the subjects signed written informed consent forms for this study. This statement explicitly states that “informed” consent was obtained from subjects’ families.

**Table 3:** Basic information of the patients used in this study.

Gender	Age	Native place	Weight	Blood Pressure(mmHg)	C677T genotype
Male	55	Lanzhou	56 kg	137/94	TT
Male	63	Lanzhou	65 kg	130/76	TT
Male	53	Lanzhou	71 kg	140/78	TT
Male	72	Lanzhou	68 kg	135/80	TT
Male	53	Lanzhou	67 kg	140/78	TT
Male	69	Lanzhou	70 kg	139/84	TT
Male	56	Lanzhou	74 kg	140/78	TT
Male	78	Lanzhou	62 kg	124/64	TT
Male	68	Lanzhou	70 kg	117/70	TT
Male	67	Lanzhou	59 kg	128/78	TT
Male	57	Lanzhou	70 kg	130/70	TT
Male	87	Lanzhou	70 kg	140/78	TT
Male	70	Lanzhou	65 kg	126/70	TT
Male	75	Lanzhou	73 kg	120/78	TT
Male	35	Lanzhou	70 kg	140/76	TT
Male	63	Lanzhou	60 kg	120/80	TT
Male	68	Lanzhou	69 kg	136/78	TT
Male	60	Lanzhou	72 kg	140/80	TT
Male	80	Lanzhou	78 kg	130/80	TT
Male	67	Lanzhou	70 kg	132/76	TT
Male	46	Lanzhou	74 kg	135/66	CC
Male	68	Lanzhou	68 kg	140/80	CC
Male	50	Lanzhou	65 kg	142/89	CC
Male	50	Lanzhou	68 kg	142/89	CC
Male	53	Lanzhou	62 kg	136/75	CC
Male	67	Lanzhou	55 kg	145/78	CC
Male	78	Lanzhou	70 kg	140/80	CC
Male	76	Lanzhou	70 kg	113/55	CC
Male	54	Lanzhou	82 kg	142/84	CC
Male	70	Lanzhou	64 kg	148/78	CC
Male	76	Lanzhou	67 kg	134/97	CC
Male	67	Lanzhou	66 kg	123/78	CC
Male	39	Lanzhou	55 kg	120/70	CC
Male	50	Lanzhou	63 kg	124/76	CC
Male	71	Lanzhou	71 kg	140/80	CC
Male	71	Lanzhou	66 kg	135/75	CC
Male	39	Lanzhou	70 kg	136/79	CC
Male	64	Lanzhou	70 kg	140/80	CC
Male	77	Lanzhou	53 kg	145/64	CC
Male	64	Lanzhou	55 kg	109/59	CC
Male	72	Lanzhou	65 kg	118/69	CC
Male	79	Lanzhou	63 kg	130/87	CC
Male	71	Lanzhou	70 kg	144/78	CC
Male	49	Lanzhou	68 kg	133/78	CC
Male	48	Lanzhou	60 kg	96/60	CC
Male	52	Lanzhou	67 kg	120/80	CC
Male	60	Lanzhou	78 kg	128/80	CC
Male	65	Lanzhou	68 kg	141/76	CC

### Treatment and quantification of the samples

The plasma of 20 patients derived from experimental group and control group were mixed and prepared for protein extraction, respectively. After protein extraction, highly abundant plasma proteins were removed by using the Proteo Extract Albumin/IgG Removal Kit (Merck, Germany). The total plasma protein concentration was measured using a Bradford protein assay kit (TIANGEN, Beijing, China) according to the manufacturer’s protocol.

### Two-dimensional electrophoresis of the plasma proteins

The hydration and loading buffers were combined with DTT, Bio-Lyte 4–6, 5–7, and the depleted plasma samples (70 μg for each experiment), and were then added into the focusing plates. Prefabricated IPG adhesive tapes were placed face down in the sample solution of the focusing plate. The adhesive tapes were placed in a PROTEAN IEF Cell electrophoresis system, and the isoelectric focusing procedure was set up (Table 4).

**Table 4:** Setup of the PROTEAN IEF Cell Isoelectric Focusing procedure.

Step	Voltage (V)	Patterns	Time	function
Hydration	50 V	Active hydration	12 hours	Hydration and loading
Step 1	250 V	Linear	30 min	Desalination
Step 2	1000 V	Fast	1 hour	Desalination
Step 3	10000 V	Linear	5 hours	Boosting
Step 4	10000 V	Fast	60,000 V/h	Focus
Step 5	500 V	Fast	At any time	Retention

Two 10% acrylamide gels were prepared. The adhesive tapes were equilibrated and were then placed face down on a long glass board with a low melting agarose molding solution on the topside. Next, the low melting agarose molding solution was frozen and the gel was transferred into an electrophoresis tank. After finishing the electrophoresis, the gel was removed and marked by a corner cut.

### Gel staining and image analysis

The gel was stained using a fast sliver stain kit. Briefly, the gel was fixed, washed, and stained using the Fast Silver Stain Kit (P0017S, Beyotime, Shanghai, China) with silver staining radiation sensitivity solution (1X) and the silver solution (1X), which was adapted to the following mass spectrometry analysis. Finally, the silver staining color-developing solution was added. This step was stopped when the protein spots were visually apparent. The gel was rinsed and preserved in ultrapure water. Images of the 2D gels were captured using the Versadoc4000images system. For experimental group and control group, three repeated experiments were performed to reduce the experimental error. Meanwhile, the protein spots were detected and optimized, and the background was reduced and additional editing was done using PDQuest8.0.1 image analysis software.

### MALDI-TOF-MS analysis: identification of the differential protein points

Seven protein spots with significant differences were selected in the gel for an API 4800 cascade time-of-flight mass spectrometer MALDI-TOF/TOF (Applied Biosystems) analysis. The spots were isolated and enzymolysis by trypsin. Subsequently, peptides are extracted, vacuum dried, and ready for analysis by mass spectrometry. The analysis was done in positive ion mode and using automatic acquisition data. The PMF’s mass spectrum scanned range was 800–3500Da, and ten peaks with a maximum strength were chosen for the second phase of mass spectrometry. After the molecular weight of the peptide fractions was accurately measured, the first and second phase mass spectrometric data obtained by integration were analyzed and identified using GPS3.6 (Applied Biosystems) and Mascot2.1 (Matrix Science) software. A t-test was used for the comparison among the groups in combination with Boolean logical operators to process. A difference was considered statistically significant when P < 0.05 (one-way ANOVA).

### Bioinformatics analysis

The standard human plasma 2D-spectrum was selected as a reference to judge the differential expression proteins (DEPs) with PDQuest8.01 software in the swiss-2DPAGE database (http://world-2dpage.expasy.org/swiss-2dpage/)^35^. The protein 3D structure was predicted by Swiss-model and was viewed usingPyMOL^36^. Sixteen differential expression proteins (DEPs) were used for the GO enrichment analysis using GOEAST (http://omicslab.genetics.ac.cn/GOEAST/index.php). A hypergeometric distribution was employed to calculate the p-value of the GOID enrichment, and a p < 1e-5 cut-off value was applied^37^. The graph size was reduced by condensing the non-significant nodes to points. The smaller the p-value is, the more significant the GO term is enriched in the dataset and the graph size was reduced by condensing the non-significant nodes to points. In addition, the DEPs were used for the preliminary signals and the metabolic pathway analysis by using the KEGG pathway mapper (Kyoto Encyclopedia of Genes and Genomes, http://www.genome.ad.jp/kegg/pathway.html) database^15–17,38^. The sequences of all of the 16 DEPs were used for BLAST analysis with the National Center for Biotechnology Information (NCBI) clusters of euKaryotic Orthologous Groups (KOG) database to obtain the KOG numbers of those proteins. A data set containing all of the KOG numbers was then used for the protein−protein interactions(PPIs) analysis by using the Search Tool for Retrieval of Interacting Genes/Proteins (STRING) database (version 9.1, http://string-db.org)^39^.

## Electronic supplementary material

Supplementary information
