# Supplementary material for: A plasma proteomics method reveals links between ischemic stroke and MTHFR C677T genotype
Source: Sci Rep. 2017 Oct 17;7:13390. doi: 10.1038/s41598-017-13542-6 (PMC5645471; doi:10.1038/s41598-017-13542-6)
Supplement: Supplementary file 1 — Supplementary information [file 41598_2017_13542_MOESM1_ESM.pdf]

Title Page

A plasma proteomics method reveals links between ischemic stroke and MTHFR C677T genotype

Zhenchang Zhang[1], Qi Yan[1], Jia Guo[1], Xueping Wang[1], Wei Yuan[1], Lei Wang[1], Lixia Chen[1], Gang Su[2.*], Manxia Wang [1,*]

[1]Department of Neurology, the Second Hospital of Lanzhou University, Lanzhou 730030, China;

[2]School of Basic Medical Sciences, Lanzhou University, Lanzhou, China

[*]Corresponding authors:

Dr. Manxia Wang

Department of Neurology, the Second Hospital of Lanzhou University, Lanzhou, China

Tel: +86-931-8942262, +86-931-8942262

No.80, Cuiyingmen, Lanzhou 730030, China

Email: wmx322@yeah.net

Dr. Gang Su

School of Basic Medical Sciences, Lanzhou University

No.222, Tianshui South Road, Lanzhou 730000, China

Tel: +86-931-8912126, Fax: +86-931-8625576

Email: sugang@lzu.edu.cn

Figure S1. Scatter diagram of differentially expressed protein spots of the MTHFR genotypes.

Figure S2. A partial enlarged diagram, 3D diagram and histogram of differentially expressed proteins of the MTHFR genotypes. A means the T/T genotypes and B means the C/C genotypes.

Table S1. Information of enriched GO terms of 16 differential expression proteins (DEPs)

Table S2. Detail information of function analysis of 16 differential expression proteins (DEPs) by using DAVID database

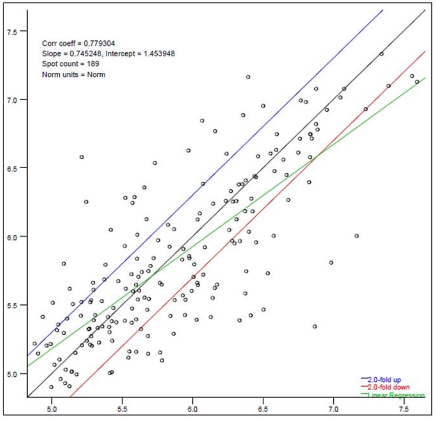
Corr coeff = 0.779304
Slope = 0.745248, Intercept = 1.453948
Spot count = 189
Norm units = Norm
2.0-fold up
2.0-fold down
Linear Regression
7.5
7.0
6.5
6.0
5.5
5.0
5.0
5.5
6.0
6.5
7.0
7.5

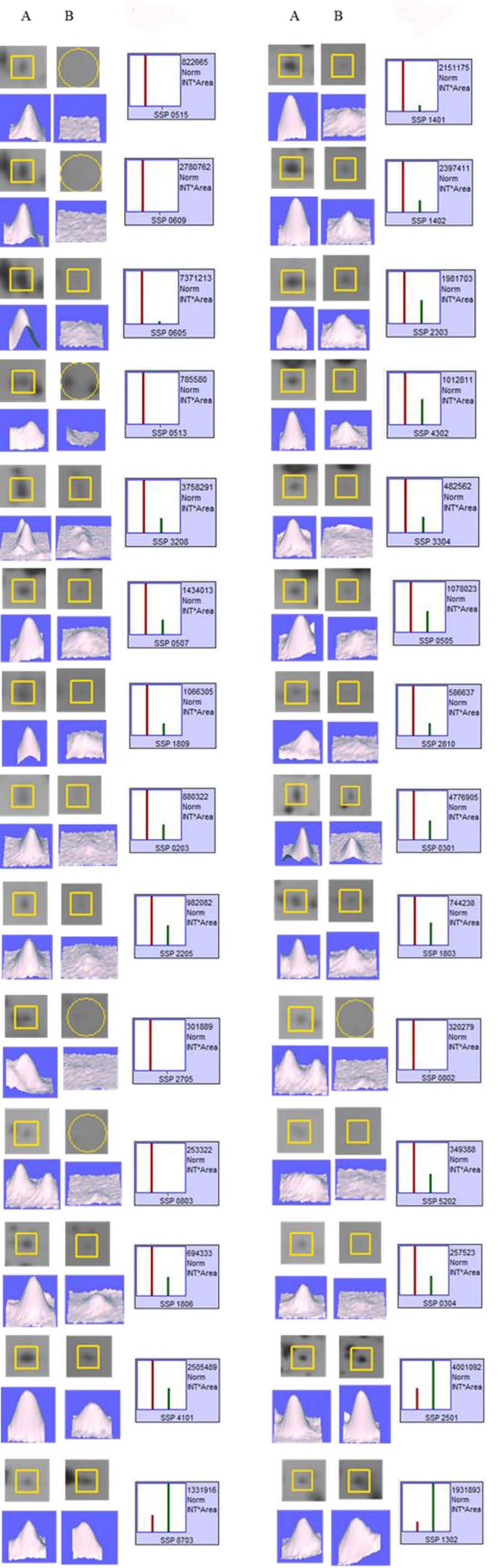

A
B
822665
Norm
INT*Area
SSP 0515
2780762
Norm
INT*Area
SSP 0609
7371213
Norm
INT*Area
SSP 0605
785580
Norm
INT*Area
SSP 0513
3758291
Norm
INT*Area
SSP 3208
1434013
Norm
INT*Area
SSP 0507
1066305
Norm
INT*Area
SSP 1809
880322
Norm
INT*Area
SSP 0203
982082
Norm
INT*Area
SSP 2205
301889
Norm
INT*Area
SSP 2705
253322
Norm
INT*Area
SSP 0803
694333
Norm
INT*Area
SSP 1806
2505489
Norm
INT*Area
SSP 4101
1331916
Norm
INT*Area
SSP 8703
A
B
2151175
Norm
INT*Area
SSP 1401
2397411
Norm
INT*Area
SSP 1402
1981703
Norm
INT*Area
SSP 2303
1012811
Norm
INT*Area
SSP 4302
482562
Norm
INT*Area
SSP 3304
1078023
Norm
INT*Area
SSP 0505
586637
Norm
INT*Area
SSP 2810
4776905
Norm
INT*Area
SSP 0301
744238
Norm
INT*Area
SSP 1803
320279
Norm
INT*Area
SSP 0802
349388
Norm
INT*Area
SSP 5202
257523
Norm
INT*Area
SSP 0304
4001092
Norm
INT*Area
SSP 2501
1931893
Norm
INT*Area
SSP 1302

TF	F2	SERPINF2	GC	AZGP1	SERPINA1	C1S	MTHFR	APCS	APOA4	TTR	CP
transferrin; Transferrins are iron binding transport proteins which can bind two Fe(3+) ions in association with the binding of an anion, usually bicarbonate. It is responsible for the transport of iron from sites of absorption and heme degradation to those of storage and utilization. Serum transferrin may also have a further role in stimulating cell proliferation	coagulation factor II (thrombin); Thrombin, which cleaves bonds after Arg and Lys, converts fibrinogen to fibrin and activates factors V, VII, VIII, XIII, and, in complex with thrombomodulin, protein C. Functions in blood homeostasis, inflammation and wound healing	serpin peptidase inhibitor, clade F (alpha-2 antiplasmin, pigment epithelium derived factor), member 2; Serine protease inhibitor. The major targets of this inhibitor are plasmin and trypsin, but it also inactivates matriptase-3/TMPRSS7 and chymotrypsin	group-specific component (vitamin D binding protein); Multifunctional protein found in plasma, ascitic fluid, cerebrospinal fluid, and urine and on the surface of many cell types. In plasma, it carries the vitamin D sterols and prevents polymerization of actin by binding its monomers. DBP associates with membrane-bound immunoglobulin on the surface of B-lymphocytes and with IgG Fc receptor on the membranes of T-lymphocytes	alpha-2-glycoprotein 1, zinc-binding; Stimulates lipid degradation in adipocytes and causes the extensive fat losses associated with some advanced cancers. May bind polyunsaturated fatty acids	serpin peptidase inhibitor, clade A (alpha-1 antiproteinase, antitrypsin), member 1	complement component 1, s subcomponent	methylenetetrahydrofolate reductase (NAD(P)H); Catalyzes the conversion of 5,10-methylenetetrahydrofolate to 5-methyltetrahydrofolate, a co-substrate for homocysteine remethylation to methionine	amyloid P component, serum; Can interact with DNA and histones and may scavenge nuclear material released from damaged circulating cells. May also function as a calcium-dependent lectin	apolipoprotein A-IV; May have a role in chylomicrons and VLDL secretion and catabolism. Required for efficient activation of lipoprotein lipase by ApoC-II; potent activator of LCAT. Apoa-IV is a major component of HDL and chylomicrons	transthyretin; Thyroid hormone-binding protein. Probably transports thyroxine from the bloodstream to the brain	ceruloplasmin (ferroxidase); Ceruloplasmin is a blue, copper-binding (6-7 atoms per molecule) glycoprotein. It has ferroxidase activity oxidizing Fe(2+) to Fe(3+) without releasing radical oxygen species. It is involved in iron transport across the cell membrane. Provides Cu(2+) ions for the ascorbate-mediated deaminase degradation of the heparan sulfate chains of GPC1. May also play a role in fetal lung development or pulmonary antioxidant defense (By similarity)
GO:0034774	GO:0043687	GO:0046330	GO:0005615	GO:0008320	GO:0005615	GO:0005515	GO:0035999	GO:0005615	GO:0005615	GO:0005615	GO:0006879
GO:0010008	GO:0008083	GO:0005615	GO:0051183	GO:0005515	GO:0007596	GO:0045087	GO:0046655	GO:0006457	GO:0046470	GO:0042562	GO:0005615
GO:0006879	GO:0005615	GO:0010951	GO:0005499	GO:0008285	GO:0010951	GO:0006956	GO:0009086	GO:0006461	GO:0007159	GO:0005737	GO:0005507
GO:0016324	GO:0048712	GO:0007596	GO:0051180	GO:0007155	GO:0002576	GO:0042802	GO:0006766	GO:0005576	GO:0033344	GO:0005179	GO:0051087
GO:0005770	GO:0008233	GO:0030199	GO:0042359	GO:0004540	GO:0032355	GO:0003824	GO:0006520	GO:0031012	GO:0005319	GO:0005515	GO:0055114
GO:0007596	GO:0007596	GO:0032967	GO:0044281	GO:0005576	GO:0005515	GO:0006958	GO:0008015	GO:0006953	GO:0034372	GO:0001523	GO:0006825
GO:0031625	GO:0005102	GO:0051918	GO:0008202	GO:0019882	GO:0001666	GO:0006508	GO:0006555	GO:0046872	GO:0005507	GO:0030198	GO:0005576
GO:0016023	GO:0007166	GO:0002576	GO:0005829	GO:0042612	GO:0010035	GO:0005576	GO:0006767	GO:0051131	GO:0034361	GO:0046982	GO:0016491
GO:0030168	GO:0032967	GO:0005515	GO:0043202	GO:0006955	GO:0014070	GO:0004252	GO:0055114	GO:0051082	GO:0005769	GO:0005576	GO:0055085
GO:0002576	GO:0051480	GO:0002034	GO:0005576		GO:0034097	GO:0005509	GO:0044281	GO:0043234	GO:0042157	GO:0006810	GO:0004322
GO:0005515	GO:0051918	GO:0045597	GO:1902118		GO:0032496		GO:0005829		GO:0034445	GO:0042572	
GO:0006826	GO:0044267	GO:0042730	GO:0003779		GO:0030168		GO:0072341		GO:0019430	GO:0007603	
GO:0005576	GO:0030193	GO:0048514			GO:0030162		GO:0004489		GO:0031210	GO:0043234	
GO:0033572	GO:0005515	GO:0045944			GO:0005576		GO:0050667		GO:0030300		
GO:0005739	GO:1900738	GO:0005577			GO:0004866				GO:0008289		
GO:0005769	GO:0009611	GO:0030168			GO:0002020				GO:0001523		
GO:0055037	GO:0042730	GO:0030162			GO:0004867				GO:0006982		
GO:0055085	GO:0014068	GO:0010033			GO:0046687				GO:0033700		
GO:0008199	GO:0070053	GO:0005576			GO:0006953				GO:0044240		
GO:0048471	GO:0003824	GO:0004866			GO:0006954				GO:0051006		
GO:0009925	GO:0008360	GO:0002020			GO:0005578				GO:0043691		
GO:0030139	GO:0006508	GO:0004867			GO:0034014				GO:0055088		
GO:0005905	GO:0007275	GO:0010757			GO:0033986				GO:0042744		
GO:0045178	GO:0030194	GO:0006953			GO:0031093				GO:0034371		
	GO:0017187	GO:0042803			GO:0010288				GO:0002227		
	GO:0005788	GO:2000049							GO:0005788		

GO:0010468	GO:0031093	GO:0010898
GO:0045861	GO:0009986	GO:0008203
GO:0007597	GO:0051496	GO:0006869
GO:0030307	GO:0048661	GO:0044281
GO:0030168	GO:0071636	GO:0016209
GO:0005796	GO:0070374	GO:0042627
GO:0010544		GO:0034364
GO:0005576		GO:0005829
GO:0001934		GO:0005576
GO:0005886		GO:0042632
GO:0004252		GO:0032374
GO:2000379		GO:0035634
GO:0050900		GO:0017127
GO:0006953		GO:0042803
GO:0051281		GO:0060228
GO:0005509		GO:0009986
GO:0008284		GO:0065005
		GO:0045723
		GO:0007603
		GO:0034375
		GO:0034378
		GO:0010873

Detail information of function analysis of 16 differential expression proteins (DEPs) by using DAVID database

Category	Term	Count	%	PValue	Genes	List Total	Pop Hits	Pop Total	Fold Enrichment	Bonferroni	Benjamini	FDR
SP_PIR_KEYWORDS	plasma	13	81.25	3.51E-26	GC, TF, APCS, HP, C1S, AHSG, APOA4, TTR, SERPINF2, F2, SERPINA3, SERPINA1, CP	16	93	19235	168.0477	3.37E-24	3.37E-24	3.86E-23
SP_PIR_KEYWORDS	acute phase	7	43.75	3.35E-14	SERPINF2, F2, SERPINA3, HP, SERPINA1, C1S, CP	16	29	19235	290.1832	3.21E-12	1.60E-12	3.68E-11
GOTERM_BP_FAT	GO:0006953~acute-phase response	7	43.75	1.33E-12	TF, APCS, SERPINF2, F2, SERPINA3, SERPINA1, AHSG	15	40	13528	157.8267	5.21E-10	5.21E-10	1.85E-09
SP_PIR_KEYWORDS	Secreted	14	87.5	1.57E-12	GC, TF, APCS, HP, AHSG, APOA4, TTR, AZGP1, SERPINF2, F2, SERPINA3, PON1, SERPINA1, CP	16	1689	19235	9.964846	1.51E-10	5.02E-11	1.73E-09
GOTERM_BP_FAT	GO:0002526~acute inflammatory response	8	50	2.77E-12	TF, APCS, SERPINF2, F2, SERPINA3, SERPINA1, C1S, AHSG	15	98	13528	73.62177	1.08E-09	5.42E-10	3.86E-09
GOTERM_CC_FAT	GO:0005615~extracellular space	12	75	1.09E-11	GC, APOA4, TF, TTR, APCS, SERPINF2, F2, PON1, HP, SERPINA1, CP, AHSG	16	685	12782	13.99489	5.86E-10	5.86E-10	1.06E-08
GOTERM_CC_FAT	GO:0005576~extracellular region	15	93.75	6.97E-11	GC, TF, APCS, HP, C1S, AHSG, APOA4, TTR, AZGP1, SERPINF2, F2, SERPINA3, PON1, SERPINA1, CP	16	2010	12782	5.961754	3.76E-09	1.88E-09	6.83E-08
SP_PIR_KEYWORDS	signal	15	93.75	1.91E-10	GC, TF, APCS, HP, C1S, AHSG, APOA4, TTR, AZGP1, SERPINF2, F2, SERPINA3, PON1, SERPINA1, CP	16	3250	19235	5.548558	1.83E-08	4.58E-09	2.10E-07
UP_SEQ_FEATURE	signal peptide	15	93.75	2.08E-10	GC, TF, APCS, HP, C1S, AHSG, APOA4, TTR, AZGP1, SERPINF2, F2, SERPINA3, PON1, SERPINA1, CP	16	3250	19113	5.513365	2.31E-08	2.31E-08	2.36E-07
GOTERM_CC_FAT	GO:0044421~extracellular region part	12	75	4.19E-10	GC, APOA4, TF, TTR, APCS, SERPINF2, F2, PON1, HP, SERPINA1, CP, AHSG	16	960	12782	9.985937	2.26E-08	7.54E-09	4.10E-07
GOTERM_BP_FAT	GO:0006952~defense response	10	62.5	1.28E-09	APOA4, TF, APCS, SERPINF2, F2, SERPINA3, HP, SERPINA1, C1S, AHSG	15	615	13528	14.6645	5.00E-07	1.67E-07	1.78E-06
SP_PIR_KEYWORDS	duplication	7	43.75	1.19E-08	GC, TF, F2, HP, C1S, CP, AHSG	16	228	19235	36.90927	1.14E-06	2.28E-07	1.31E-05
GOTERM_BP_FAT	GO:0006954~inflammatory response	8	50	1.29E-08	TF, APCS, SERPINF2, F2, SERPINA3, SERPINA1, C1S, AHSG	15	325	13528	22.19979	5.03E-06	1.26E-06	1.79E-05

SP_PIR_KEYWORDS	liver	5	31.25	1.06E-07	GC, TTR, F2, HP, AHSG	16	59	19235	101.8803	1.02E-05	1.70E-06	1.17E-04
UP_SEQ_FEATURE	glycosylation site:N-linked (GlcNAc...)	14	87.5	1.48E-07	GC, TF, APCS, HP, C1S, AHSG, TTR, AZGP1, SERPINF2, F2, SERPINA3, PON1, SERPINA1, CP	16	4129	19113	4.050345	1.64E-05	8.20E-06	1.67E-04
SP_PIR_KEYWORDS	glycoprotein	14	87.5	2.39E-07	GC, TF, APCS, HP, C1S, AHSG, TTR, AZGP1, SERPINF2, F2, SERPINA3, PON1, SERPINA1, CP	16	4318	19235	3.897783	2.29E-05	3.28E-06	2.63E-04
GOTERM_BP_FAT	GO:0009611~response to wounding	8	50	3.68E-07	TF, APCS, SERPINF2, F2, SERPINA3, SERPINA1, C1S, AHSG	15	530	13528	13.61308	1.44E-04	2.88E-05	5.11E-04
UP_SEQ_FEATURE	disulfide bond	11	68.75	7.06E-06	GC, AZGP1, TF, APCS, SERPINF2, F2, PON1, HP, C1S, CP, AHSG	16	2819	19113	4.661294	7.83E-04	2.61E-04	0.007985
SP_PIR_KEYWORDS	disulfide bond	11	68.75	9.33E-06	GC, AZGP1, TF, APCS, SERPINF2, F2, PON1, HP, C1S, CP, AHSG	16	2924	19235	4.522593	8.95E-04	1.12E-04	0.010271
KEGG_PATHWAY	hsa04610:Complement and coagulation cascades	4	25	2.35E-05	SERPINF2, F2, SERPINA1, C1S	6	69	5085	49.13043	1.64E-04	1.64E-04	0.012094
SP_PIR_KEYWORDS	serine proteinase inhibitor	3	18.75	1.30E-04	SERPINF2, SERPINA3, SERPINA1	16	22	19235	163.9347	0.012398	0.001385	0.143026
SP_PIR_KEYWORDS	amyloid	3	18.75	1.69E-04	TTR, APCS, SERPINA3	16	25	19235	144.2625	0.01605	0.001617	0.185462
GOTERM_CC_FAT	GO:0034364~high-density lipoprotein particle	3	18.75	3.80E-04	APOA4, PON1, HP	16	25	12782	95.865	0.020297	0.005113	0.371322
GOTERM_BP_FAT	GO:0006879~cellular iron ion homeostasis	3	18.75	4.55E-04	TF, HP, CP	15	31	13528	87.27742	0.162885	0.029197	0.630144
GOTERM_MF_FAT	GO:0004866~endopeptidase inhibitor activity	4	25	5.63E-04	SERPINF2, SERPINA3, SERPINA1, AHSG	16	145	12983	22.38448	0.045101	0.045101	0.599446
GOTERM_BP_FAT	GO:0010033~response to organic substance	6	37.5	5.67E-04	TF, APCS, SERPINF2, SERPINA1, C1S, AHSG	15	721	13528	7.505132	0.198983	0.031199	0.78576
INTERPRO	IPR000215:Protease inhibitor I4, serpin	3	18.75	5.79E-04	SERPINF2, SERPINA3, SERPINA1	16	40	16659	78.08906	0.029653	0.029653	0.560833
GOTERM_BP_FAT	GO:0055072~iron ion homeostasis	3	18.75	6.14E-04	TF, HP, CP	15	36	13528	75.15556	0.213521	0.029577	0.850348

GOTERM_MF_FAT	GO:0030414~peptidase inhibitor activity	4	25	6.58E-04	SERPINF2, SERPINA3, SERPINA1, AHSG	16	153	12983	21.21405	0.052548	0.026628	0.700778
GOTERM_BP_FAT	GO:0042592~homeostatic process	6	37.5	6.84E-04	APOA4, TF, F2, SERPINA3, HP, CP	15	751	13528	7.205326	0.234753	0.029291	0.946777
GOTERM_CC_FAT	GO:0032994~protein-lipid complex	3	18.75	7.48E-04	APOA4, PON1, HP	16	35	12782	68.475	0.039597	0.008048	0.730297
GOTERM_CC_FAT	GO:0034358~plasma lipoprotein particle	3	18.75	7.48E-04	APOA4, PON1, HP	16	35	12782	68.475	0.039597	0.008048	0.730297
SP_PIR_KEYWORDS	disease mutation	7	43.75	8.25E-04	TF, TTR, MTHFR, SERPINF2, F2, SERPINA3, SERPINA1	16	1591	19235	5.289323	0.076201	0.00718	0.905227
SMART	SM00093:SERPIN	3	18.75	0.001015189	SERPINF2, SERPINA3, SERPINA1	12	40	9079	56.74375	0.013117	0.013117	0.670203
PIR_SUPERFAMILY	PIRSF001630:serpin	3	18.75	0.00146039	SERPINF2, SERPINA3, SERPINA1	14	33	7396	48.02597	0.021683	0.021683	1.010947
GOTERM_BP_FAT	GO:0030005~cellular di-, tri-valent inorganic cation homeostasis	4	25	0.001480261	TF, F2, HP, CP	15	227	13528	15.89192	0.43966	0.056276	2.038303
GOTERM_BP_FAT	GO:0048878~chemical homeostasis	5	31.25	0.001499417	APOA4, TF, F2, HP, CP	15	512	13528	8.807292	0.443847	0.05194	2.064425
UP_SEQ_FEATURE	region of interest:RCL	2	12.5	0.001569037	SERPINA3, SERPINA1	16	2	19113	1194.563	0.159955	0.042639	1.761393
GOTERM_BP_FAT	GO:0055066~di-, tri-valent inorganic cation homeostasis	4	25	0.001716138	TF, F2, HP, CP	15	239	13528	15.094	0.489103	0.054428	2.359519
SP_PIR_KEYWORDS	Serine protease inhibitor	3	18.75	0.001731757	SERPINF2, SERPINA3, SERPINA1	16	80	19235	45.08203	0.153286	0.01377	1.890899
GOTERM_BP_FAT	GO:0051240~positive regulation of multicellular organismal process	4	25	0.001821001	TF, SERPINF2, F2, AHSG	15	244	13528	14.7847	0.509662	0.053345	2.502006
GOTERM_BP_FAT	GO:0030003~cellular cation homeostasis	4	25	0.002042643	TF, F2, HP, CP	15	254	13528	14.20262	0.550442	0.055506	2.802537

GOTERM_BP_FAT	GO:0034444~regulation of plasma lipoprotein oxidation	2	12.5	0.002068787	APOA4, PON1	15	2	13528	901.8667	0.555023	0.052551	2.83793
GOTERM_BP_FAT	GO:0034445~negative regulation of plasma lipoprotein oxidation	2	12.5	0.002068787	APOA4, PON1	15	2	13528	901.8667	0.555023	0.052551	2.83793
GOTERM_BP_FAT	GO:0055080~cation homeostasis	4	25	0.00286318	TF, F2, HP, CP	15	286	13528	12.61352	0.674082	0.067671	3.907664
UP_SEQ_FEATURE	domain:Peptidase S1	3	18.75	0.003051976	F2, HP, C1S	16	106	19113	33.80837	0.287722	0.065606	3.400087
SP_PIR_KEYWORDS	protease inhibitor	3	18.75	0.003070316	SERPINF2, SERPINA3, SERPINA1	16	107	19235	33.70619	0.25562	0.022452	3.330127
GOTERM_BP_FAT	GO:0034442~regulation of lipoprotein oxidation	2	12.5	0.003101689	APOA4, PON1	15	3	13528	601.2444	0.703185	0.068957	4.226701
GOTERM_BP_FAT	GO:0034443~negative regulation of lipoprotein oxidation	2	12.5	0.003101689	APOA4, PON1	15	3	13528	601.2444	0.703185	0.068957	4.226701
GOTERM_MF_FAT	GO:0004857~enzyme inhibitor activity	4	25	0.003362458	SERPINF2, SERPINA3, SERPINA1, AHSG	16	270	12983	12.0213	0.241328	0.087951	3.534253
INTERPRO	IPR001314:Peptidase S1A, chymotrypsin	3	18.75	0.003843773	F2, HP, C1S	16	104	16659	30.03425	0.181483	0.095281	3.672535
INTERPRO	IPR001254:Peptidase S1 and S6, chymotrypsin/Hap	3	18.75	0.00475704	F2, HP, C1S	16	116	16659	26.92726	0.219606	0.079329	4.527125
GOTERM_MF_FAT	GO:0004867~serine-type endopeptidase inhibitor activity	3	18.75	0.004911659	SERPINF2, SERPINA3, SERPINA1	16	92	12983	26.45992	0.332188	0.09601	5.12418
SP_PIR_KEYWORDS	polymorphism	15	93.75	0.005210389	GC, TF, APCS, HP, C1S, AHSG, APOA4, TTR, MTHFR, SERPINF2, F2, SERPINA3, PON1, SERPINA1, CP	16	11550	19235	1.561282	0.394381	0.035188	5.591311
GOTERM_BP_FAT	GO:0032844~regulation of homeostatic process	3	18.75	0.005995745	TF, F2, AHSG	15	114	13528	23.73333	0.904764	0.12246	8.020355
GOTERM_BP	GO:0006873~cellular ion	4	25	0.006081	TF, F2, HP, CP	15	374	13528	9.645633	0.907915	0.11797	8.130353

_FAT	homeostasis			3									
GOTERM_BP _FAT	GO:0050748~negative regulation of lipoprotein metabolic process	2	12.5	0.006194 443	APOA4, PON1	15	6	13528	300.6222	0.911924	0.114389	8.275631
GOTERM_BP _FAT	GO:0055082~cellular chemical homeostasis	4	25	0.006356 059	TF, F2, HP, CP	15	380	13528	9.493333	0.917351	0.111945	8.482782
GOTERM_BP _FAT	GO:0050801~ion homeostasis	4	25	0.007789 64	TF, F2, HP, CP	15	409	13528	8.820212	0.953003	0.129759	10.30135
GOTERM_BP _FAT	GO:0050746~regulation of lipoprotein metabolic process	2	12.5	0.008251 326	APOA4, PON1	15	8	13528	225.4667	0.960823	0.131384	10.87984
SMART	SM00020:Tryp_SPc	3	18.75	0.008255 726	F2, HP, C1S	12	116	9079	19.56681	0.102166	0.052459	5.34059
UP_SEQ_FEA TURE	sequence variant	15	93.75	0.009087 204	GC, TF, APCS, HP, C1S, AHSG, APOA4, TTR, MTHFR, SERPINF2, F2, SERPINA3, PON1, SERPINA1, CP	16	11992	19113	1.494199	0.636978	0.155391	9.81535
GOTERM_CC _FAT	GO:0034366~spherical high-density lipoprotein particle	2	12.5	0.009352 283	PON1, HP	16	8	12782	199.7188	0.397941	0.08109	8.794157
GOTERM_BP _FAT	GO:0030194~positive regulation of blood coagulation	2	12.5	0.010304 256	SERPINF2, F2	15	10	13528	180.3733	0.982575	0.155275	13.41049
GOTERM_BP _FAT	GO:0019725~cellular homeostasis	4	25	0.011134 903	TF, F2, HP, CP	15	466	13528	7.741345	0.987452	0.160649	14.41534
GOTERM_BP _FAT	GO:0032101~regulation of response to external stimulus	3	18.75	0.011386 303	SERPINF2, F2, AHSG	15	159	13528	17.01635	0.988639	0.158202	14.71733
GOTERM_MF _FAT	GO:0004252~serine-type endopeptidase activity	3	18.75	0.013264 395	F2, HP, C1S	16	154	12983	15.80722	0.665447	0.196672	13.29458
GOTERM_BP _FAT	GO:0050820~positive regulation of coagulation	2	12.5	0.013376 253	SERPINF2, F2	15	13	13528	138.7487	0.994833	0.177178	17.07302
GOTERM_MF _FAT	GO:0008289~lipid binding	4	25	0.013793 487	GC, APOA4, AZGP1, PON1	16	450	12983	7.212778	0.679841	0.172895	13.78997
SP_PIR_KEY	hdl	2	12.5	0.013950	APOA4, PON1	16	18	19235	133.5764	0.740414	0.085988	14.33543

WORDS				378								
SP_PIR_KEYWORDS	gamma-carboxyglutamic acid	2	12.5	0.014720048	TTR, F2	16	19	19235	126.5461	0.759161	0.085133	15.06902
GOTERM_MF_FAT	GO:0008236~serine-type peptidase activity	3	18.75	0.017455779	F2, HP, C1S	16	178	12983	13.67591	0.764021	0.186401	17.14927
SP_PIR_KEYWORDS	iron transport	2	12.5	0.017793124	TF, HP	16	23	19235	104.538	0.821563	0.096414	17.94146
GOTERM_MF_FAT	GO:0017171~serine hydrolase activity	3	18.75	0.017827658	F2, HP, C1S	16	180	12983	13.52396	0.771234	0.168382	17.48365
GOTERM_CC_FAT	GO:0030141~secretory granule	3	18.75	0.018358581	TF, SERPINF2, SERPINA1	16	180	12782	13.31458	0.632333	0.133193	16.60002
GOTERM_BP_FAT	GO:0032374~regulation of cholesterol transport	2	12.5	0.020509846	APOA4, PON1	15	20	13528	90.18667	0.999697	0.251275	25.03055
GOTERM_BP_FAT	GO:0032371~regulation of sterol transport	2	12.5	0.020509846	APOA4, PON1	15	20	13528	90.18667	0.999697	0.251275	25.03055
GOTERM_BP_FAT	GO:0046470~phosphatidylcholine metabolic process	2	12.5	0.021525012	APOA4, PON1	15	21	13528	85.89206	0.999798	0.254265	26.10354
GOTERM_BP_FAT	GO:0051050~positive regulation of transport	3	18.75	0.02160758	F2, PON1, AHSG	15	223	13528	12.13274	0.999805	0.247765	26.19018
GOTERM_MF_FAT	GO:0002020~protease binding	2	12.5	0.021739929	SERPINF2, SERPINA1	16	19	12983	85.41447	0.835087	0.181482	20.92812
SP_PIR_KEYWORDS	calcium	4	25	0.022653544	APCS, F2, PON1, C1S	16	803	19235	5.988481	0.889169	0.115036	22.30466
SP_PIR_KEYWORDS	extracellular protein	2	12.5	0.023912455	AZGP1, SERPINF2	16	31	19235	77.56048	0.902069	0.115107	23.39984
GOTERM_MF_FAT	GO:0008233~peptidase activity	4	25	0.026292131	F2, HP, SERPINA1, C1S	16	574	12983	5.654617	0.8875	0.196261	24.77161
GOTERM_BP_FAT	GO:0032368~regulation of lipid transport	2	12.5	0.030617642	APOA4, PON1	15	30	13528	60.12444	0.999995	0.32444	35.09817
SP_PIR_KEY	blood coagulation	2	12.5	0.033780	F2, SERPINA1	16	44	19235	54.64489	0.963079	0.152063	31.51032

WORDS				532								
UP_SEQ_FEATURE	domain:Sushi 2	2	12.5	0.033992771	HP, C1S	16	44	19113	54.2983	0.978481	0.422128	32.38838
UP_SEQ_FEATURE	domain:Sushi 1	2	12.5	0.033992771	HP, C1S	16	44	19113	54.2983	0.978481	0.422128	32.38838
GOTERM_BP_FAT	GO:0042439~ethanolamine and derivative metabolic process	2	12.5	0.034633572	APOA4, PON1	15	34	13528	53.05098	0.999999	0.349934	38.73774
UP_SEQ_FEATURE	site:Reactive bond	2	12.5	0.034752649	SERPINA3, SERPINA1	16	45	19113	53.09167	0.980281	0.387846	32.98784
SP_PIR_KEYWORDS	transport	5	31.25	0.035545852	GC, APOA4, TF, TTR, CP	16	1670	19235	3.599364	0.969024	0.152491	32.87599
GOTERM_BP_FAT	GO:0030193~regulation of blood coagulation	2	12.5	0.036635739	SERPINF2, F2	15	36	13528	50.1037	1	0.357398	40.48064
GOTERM_BP_FAT	GO:0032846~positive regulation of homeostatic process	2	12.5	0.04062851	F2, AHSG	15	40	13528	45.09333	1	0.379348	43.81982
SP_PIR_KEYWORDS	serine proteinase	2	12.5	0.041307913	F2, C1S	16	54	19235	44.52546	0.982574	0.168132	37.16311
GOTERM_BP_FAT	GO:0050818~regulation of coagulation	2	12.5	0.041624299	SERPINF2, F2	15	41	13528	43.9935	1	0.378089	44.62507
UP_SEQ_FEATURE	metal ion-binding site:Calcium 2	2	12.5	0.042320804	APCS, PON1	16	55	19113	43.43864	0.99177	0.413348	38.69931
SP_PIR_KEYWORDS	sushi	2	12.5	0.042806806	HP, C1S	16	56	19235	42.93527	0.985004	0.166906	38.23674
GOTERM_CC_FAT	GO:0031093~platelet alpha granule lumen	2	12.5	0.047074281	SERPINF2, SERPINA1	16	41	12782	38.96951	0.926007	0.277815	37.64793
INTERPRO	IPR000436:Sushi/SCR/CCP	2	12.5	0.049274029	HP, C1S	16	56	16659	37.18527	0.927743	0.481535	38.79406
INTERPRO	IPR016060:Complement control module	2	12.5	0.050132964	HP, C1S	16	57	16659	36.53289	0.931061	0.414276	39.32919

GOTERM_CC_FAT	GO:0060205~cytoplasmic membrane-bounded vesicle lumen	2	12.5	0.050436227	SERPINF2, SERPINA1	16	44	12782	36.3125	0.938863	0.266931	39.76983
GOTERM_CC_FAT	GO:0031983~vesicle lumen	2	12.5	0.05267137	SERPINF2, SERPINA1	16	46	12782	34.7337	0.946169	0.253373	41.14443
GOTERM_CC_FAT	GO:0031012~extracellular matrix	3	18.75	0.06051445	TF, SERPINA1, AHSG	16	345	12782	6.946739	0.965639	0.263937	45.7479
GOTERM_CC_FAT	GO:0031091~platelet alpha granule	2	12.5	0.063773619	SERPINF2, SERPINA1	16	56	12782	28.53125	0.971518	0.256615	47.56379
SMART	SM00032:CCP	2	12.5	0.065829792	HP, C1S	12	56	9079	27.02083	0.587392	0.255532	36.29084
GOTERM_MF_FAT	GO:0004175~endopeptidase activity	3	18.75	0.068198414	F2, HP, C1S	16	375	12983	6.4915	0.996948	0.409364	52.98055
UP_SEQ_FEATURE	glycosylation site:O-linked (GalNAc...)	2	12.5	0.069844109	TF, AHSG	16	92	19113	25.96875	0.999677	0.552319	55.9301
GOTERM_BP_FAT	GO:0000041~transition metal ion transport	2	12.5	0.071055462	TF, CP	15	71	13528	25.40469	1	0.55091	64.10793
GOTERM_MF_FAT	GO:0005507~copper ion binding	2	12.5	0.076861011	APOA4, CP	16	69	12983	23.51993	0.998581	0.421027	57.44575
SP_PIR_KEYWORDS	calcium binding	2	12.5	0.077407428	F2, C1S	16	103	19235	23.34345	0.999563	0.275498	58.82622
GOTERM_BP_FAT	GO:0030001~metal ion transport	3	18.75	0.081699275	TF, F2, CP	15	465	13528	5.818495	1	0.593706	69.42112
GOTERM_BP_FAT	GO:0032268~regulation of cellular protein metabolic process	3	18.75	0.084450071	APOA4, F2, PON1	15	474	13528	5.708017	1	0.596607	70.67021
GOTERM_BP_FAT	GO:0007586~digestion	2	12.5	0.090207572	APOA4, SERPINA3	15	91	13528	19.82125	1	0.612412	73.13284
INTERPRO	IPR018114:Peptidase S1/S6, chymotrypsin/Hap, active site	2	12.5	0.090520389	F2, C1S	16	105	16659	19.83214	0.992802	0.560588	60.22163

GOTERM_BP_FAT	GO:0006576~biogenic amine metabolic process	2	12.5	0.095881303	APOA4, PON1	15	97	13528	18.59519	1	0.626661	75.37069
